# LC/MS-Based Quantitative Proteomic Analysis of Paraffin-Embedded Archival Melanomas Reveals Potential Proteomic Biomarkers Associated with Metastasis

**DOI:** 10.1371/journal.pone.0004430

**Published:** 2009-02-16

**Authors:** Sharon K. Huang, Marlene M. Darfler, Michael B. Nicholl, Jinsam You, Kerry G. Bemis, Tony J. Tegeler, Mu Wang, Jean-Pierre Wery, Kelly K. Chong, Linhda Nguyen, Richard A. Scolyer, Dave S. B. Hoon

**Affiliations:** 1 Department of Molecular Oncology, John Wayne Cancer Institute at Saint John's Health Center, Santa Monica, California, United States of America; 2 Expression Pathology, Inc., Gaithersburg, Maryland, United States of America; 3 Monarch LifeSciences LLC, Indianapolis, Indiana, United States of America; 4 Sydney Melanoma Unit, Sydney Cancer Center, Royal Prince Alfred Hospital, Camperdown, Australia; Cleveland Clinic, United States of America

## Abstract

**Background:**

Melanoma metastasis status is highly associated with the overall survival of patients; yet, little is known about proteomic changes during melanoma tumor progression. To better understand the changes in protein expression involved in melanoma progression and metastasis, and to identify potential biomarkers, we conducted a global quantitative proteomic analysis on archival metastatic and primary melanomas.

**Methodology and Findings:**

A total of 16 metastatic and 8 primary cutaneous melanomas were assessed. Proteins were extracted from laser captured microdissected formalin fixed paraffin-embedded archival tissues by liquefying tissue cells. These preparations were analyzed by a LC/MS-based label-free protein quantification method. More than 1500 proteins were identified in the tissue lysates with a peptide ID confidence level of >75%. This approach identified 120 significant changes in protein levels. These proteins were identified from multiple peptides with high confidence identification and were expressed at significantly different levels in metastases as compared with primary melanomas (q-Value<0.05).

**Conclusions and Significance:**

The differentially expressed proteins were classified by biological process or mapped into biological system networks, and several proteins were implicated by these analyses as cancer- or metastasis-related. These proteins represent potential biomarkers for tumor progression. The study successfully identified proteins that are differentially expressed in formalin fixed paraffin-embedded specimens of metastatic and primary melanoma.

## Introduction

The potential for primary melanomas to metastasize increases with the depth of invasion. The diagnosis of distant metastatic cutaneous melanoma is usually associated with poor prognosis. Currently, no single protein marker reliably predicts disease outcome in cutaneous melanoma. Understanding the mechanisms of metastasis and identifying proteomic biomarkers associated with melanoma progression and metastasis may aid in developing treatment strategies. In recent years, many studies have investigated gene expression signatures based on mRNA levels in melanomas [1], [2], [3]. However, changes at the mRNA level may not always directly correlate to changes at the protein level; translational and post-translational alterations also affect tumor progression and metastasis. Proteomics screening tools may identify relevant and significant changes in protein expression in relation to early and advance stage cancers.

Two-dimensional polyacrylamide gel electrophoresis (2D-PAGE) coupled with tandem mass spectrometry (MS/MS) has been the principal tool for proteomic analysis of melanoma tissue [4], [5], but little data has been reported about the proteomic expression profiles of primary versus metastatic melanoma tissues. The ion intensity-based, label-free quantitative proteomics (LFQP) approach has gained popularity as mass spectrometer performance has significantly improved [6], [7], [8]. LFQP provides a powerful tool to resolve and identify thousands of proteins from a complex biological sample. Proteins are digested with a protease, and the peptide mixture is analyzed by liquid chromatography-mass spectrometry (LC/MS) and liquid chromatography with tandem mass spectrometry (LC/MS/MS); the relative protein abundance is determined by chromatographic peak intensity measurement. The LFQP approach is rapid and more sensitive than many other proteomic methods, and it increases the protein dynamic range 3- to 4-fold as compared to 2D-PAGE. This method can also be automated for large-scale proteome analysis.

To identify the mechanisms and proteomic biomarkers of melanoma metastasis, we used an LC/MS-based label-free protein quantification method to profile the global protein expression of microdissected primary and metastatic melanoma formalin fixed paraffin-embedded (FFPE) archival tissues. Proteins were extracted using reagents and protocols that yield a product suitable for MS analysis [9], [10]. The overall objective was to determine if proteomic profiling differences could be identified in FFPE tissue specimens of primary and metastatic cutaneous melanomas.

## Methods

### Tissue Processing

Formalin-fixed tissue blocks from 8 primary (five AJCC stage II and three AJCC stage III) and 16 metastatic melanomas (5 AJCC stage III lymph nodes and AJCC stage IV: 4 lung, 4 brain, 1 colon, 1 adrenal gland, and 1 extremity) were chosen for analysis. All cutaneous melanomas were identified by standard histopathology techniques. Five µm thick tissue sections were stained with hematoxylin and eosin to identify an 8-mm^2^ area containing defined melanoma cells for dissection. For tissue isolation and preparation of analyzable protein, the manufacturer's recommendations were followed (Expression Pathology, Inc., Gaithersburg, MD). In brief, a single 10-µm tissue section was cut from each tissue block, treated with xylene to remove paraffin, rehydrated through a series of graded ethanol solutions and distilled water, and stained with hematoxylin. An area 8 mm^2^ in size, corresponding to approximately 30,000 cells from the relevant region, was procured by microdissection for processing. Informed patient consents for all studies were approved by the human subjects IRB of Saint John's Health Center and JWCI. All of the consents are written and verbally explained to the patients. Patients' specimens were de-identified and run in a double-blinded manner.

### Sample Preparation

Collected microdissected tissue was processed with the Liquid Tissue® MS Protein Prep Kit (Expression Pathology, Inc.). Briefly, the tissue was suspended in 20 µl of Liquid Tissue Buffer, incubated at 95°C for 90 min, and then cooled on ice for 2 min. Trypsin (1 µg) was added, and then the tissue was incubated at 37°C overnight. The sample was then heated for 5 min at 95°C to inactivate the trypsin. After the total amount of extracted protein was measured by a micro BCA (bicinchoninic acid) protein assay (PIERCE, Rockford, IL), DTT was added for a final concentration of 10 mM. The sample was stored at −20°C until LC/MS/MS analysis.

### LC/MS/MS Analysis

Trypsin-digested samples (1 µg each) were injected onto an Agilent 1100 nano-HPLC system (Agilent Technologies, Inc., Santa Clara, CA) with a C18 capillary column in random order. Peptides were eluted with a linear gradient from 5 to 45% acetonitrile developed over 120 min at a flow rate of 500 nL/min, and effluent was electro-sprayed into the LTQ mass spectrometer (Thermo Fisher Scientific Inc., Waltham, MA). Data was collected in the “Triple-Play” (MS scan, Zoom scan, and MS/MS scan) mode. The acquired data were filtered and analyzed by a proprietary algorithm [11]. Database searches against the International Protein Index (IPI) human database (European Bioinformatics Institute, 2005) and the Non-Redundant-homo sapiens database (National Center for Biotechnology Information, 2005) were carried out using both the X!Tandem [12] and SEQUEST [13] algorithms. Protein quantification was carried out using the same algorithm described above [11]. Briefly, when the raw files were acquired from the LTQ mass spectrometer, all extracted ion chromatograms (XICs) were aligned by retention time. To be used in the protein quantification procedure, each aligned peak must have a matched precursor ion, charge state, fragment ions (MS/MS data) and retention time (within a one-minute window). After alignment, the area-under-the-curve (AUC) for each individually aligned peak from each sample was measured, normalized, and compared for relative abundance as previously described [11].

After obtaining the list of proteins that were differentially expressed in primary and metastatic samples from the LC/MS quantitative analysis, a corresponding gene list was created and input for bioinformatics analysis.

### Biostatistics Analysis

Since the data has multiple sources of random variation, such as biological samples and replicates, a Linear Mixed Model (a generalization of an ANOVA, Analysis of Variance) was used for biostatistics analysis in this study. This model, especially when applied to complex experimental designs, cannot be handled by introductory methods such as t-tests. Also, the exact scale of the protein expression used in the model can make a difference in the sensitivity. In general, there is usually a large technical variation introduced by the act of ‘measurement’ in many ‘omics’ studies. Randomization of measurement order and normalization of the data will eliminate the technical bias. We used a statistically based quantile normalization method for data normalization [14]. Because ‘omics’ measures of expression are usually on an arbitrary scale, we chose to evaluate ratios or their equivalent differences on a log scale [15]. Log base 2 was chosen because a unit difference on the log scale is equivalent to a two-fold change.

A byproduct of the Linear Mixed Model is a p-Value or measure of significance for an observed change in protein expression (signal). The p-Value estimates the proportion of times a change that large will be observed if in fact there is no real change (the False Positive Rate). All the p-Values were then transformed into q-Values that estimate the False Discovery Rate (FDR) [16].

For each protein a separate analysis of variance (ANOVA) model is fit:
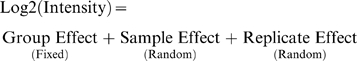

*Log2(Intensity)* is the protein intensity based on the weighted average of the quantile normalized log base 2 peptide intensities with the same protein identification. *Group Effect* refers to the fixed effects (not random) caused by the experimental conditions or treatments that are being compared. *Sample Effect* (nested within group) refers to the random effects from individual biological samples. It also includes the random effects from the individual sample preparations. *Replicate Effect* (nested within sample) refers to the random effects from replicate injections from the same sample preparation.

When an ANOVA model has two or more random effects (sample and replicate in this data) and at least one fixed effect (group in this data) it is referred to as a Mixed Model. For each protein these models were fit using PROC MIXED in SAS software (version 9) (SAS Institute Inc., Cary, NC). The REML method was used as a fit mechanism and degrees of freedom were computed using the Satterthwaite method. The RANDOM statement was used to model the covariance with the NOBOUND parameter option in the PROC statement.

### Immunohistochemistry Analysis

Selective protein expressions were assessed by immunohistochemistry (IHC) on FFPE melanoma tissues. Five-µm sections were cut from FFPE archival tissue blocks and incubated overnight at 50°C for IHC preparation. The sections were then deparaffinized in xylene and rehydrated in ethanol. All IHC staining was conducted with the CSA II kit (CSA II, Biotin-Free Tyramide Signal Amplication System; Dakocytomation) as described previously [17]. To perform heat-induced antigen retrieval, sections were placed into 10 mM citrate buffer (pH 6.0) and heated to 100°C for 20 min. Endogenous peroxidase function was quenched with peroxidase blocking solution. Nonspecific protein binding sites were blocked with serum-free protein blocking solution. Sections were incubated with anti-STAT1 mAb (BD Transduction, San Jose, CA) at 1∶400 dilution in PBS for 30 min at room temperature, or anti-MIF mAb (Abcam, Cambridge, MA) at 1∶100 dilution in PBS, anti-pyruvate kinase (PKM1/M2) (Abcam) at 1∶50 dilution in antibody diluent (Dakocytomation) with 1% BSA, or prediluted anti-HSP90α (Hsp86 antibody, Abcam) overnight at 4°C. Negative control slides were incubated under the same conditions with universal negative control-mouse antibody or universal negative control-rabbit (Dakocytomation), respectively. After washing, tissue sections stained with mouse monoclonal primary antibodies were incubated for 15 min at room temperature with horseradish peroxidase conjugated anti-mouse Ig antibodies. CSA II Rabbit Link (DakoCytomation) was used for the detection of rabbit primary antibodies. The amplification system provided with the CSA II kit was applied as directed in the manufacturer's instructions. Slides were developed with DAB. Sections were counterstained with half-strength Gill's hematoxylin (Fisher Scientific) for 30 sec, then dehydrated and mounted. Sections were evaluated and photographed using a Nikon Eclipse Ti microscope system.

### Pathway Networks Analysis

The differentially expressed proteins were mapped into biological networks by using a manually curated proprietary database (MetaCore™, GeneGo, St. Joseph, MI), a pathway analysis tool. Gene symbols of differentially expressed proteins were uploaded into the database. For enrichment analysis, gene IDs of the uploaded files were matched with gene IDs in functional ontologies in MetaCore™ [18], that included canonical pathway maps (GeneGo maps), GeneGo cellular processes, Gene Ontology (GO) cellular process and diseases categories. Canonical pathway maps represent a set of about 500 signaling and metabolic maps covering human biology. For network analysis, analyze networks (AN), transcription regulation and direct interactions algorithms were used. The analyze networks algorithm, a variant of the shortest paths algorithm, uses relative enrichment and relative saturation to generate subnetworks prioritized by their number of canonical pathways [19].

## Results

### Proteomic Profiling of FFPE Melanoma Tissue

Proteins were extracted from microdissected melanoma FFPE tissues and the global protein expression profile in metastases was compared to that in primary tissues. MS analysis results are summarized in **Table 1**. Of the 1557 proteins identified and quantified, 555 proteins were identified with high confidence (Priority 1). The identified proteins were found in every cell compartment and appeared to cover a broad range of biological functions. Of the 555 Priority 1 proteins, 120 proteins were differentially expressed (q<0.05) in primary and metastatic melanomas. The q-value, an adjusted p-value, was used to estimate the False Discovery Rate (FDR) [20]. A False Discovery is a when protein is falsely declared to be changed. The significance threshold sets the FDR at less than 5%. The replicate median percentage coefficient of variation (%CV) for the Priority 1 proteins was 7.6%, and the combined replicate plus sample median %CV was 15%.

**Table 1:** LC/MS-based Label-free Protein Quantification Method.

Protein Priority	Peptide ID Confidence	Multiple Sequences	Number of Proteins	Number Significant Changes	Maximum Foldchange*	Median %CV**	Median %CV***
1	HIGH	YES	555	120	2.8	7.6	15
2	HIGH	NO	480	133	6.8	14	25.3
3	LOW	YES	8	3	2	9.5	24.4
4	LOW	NO	514	227	7.1	13	32.9
*Overall*			*1557*	*483*	*7.1*	*11.7*	*22.6*

* Absolute Fold Change.

** replicate.

*** replicate+sample.

### Characterization of Identified Proteins

**Figure 1:**
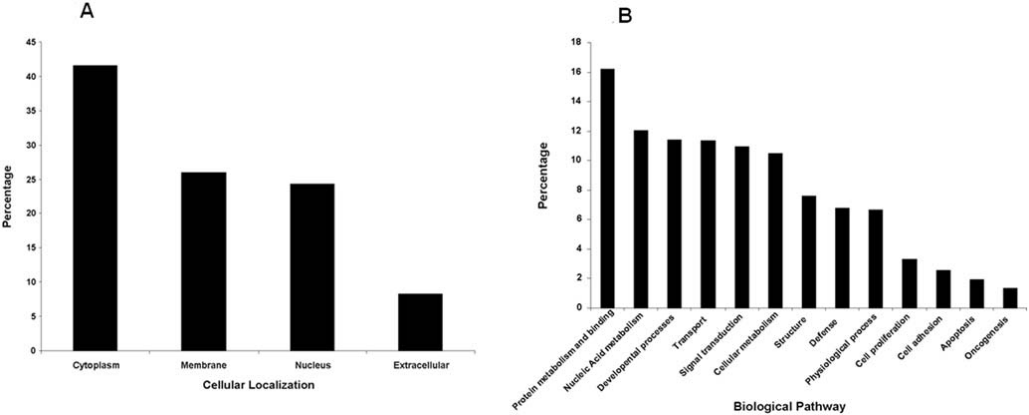
Location and functional distribution of proteins isolated and identified from melanoma FFPE tissue. Proteins are categorized by A) cellular origin and, B) biological process, based on the Gene Ontology database.

To investigate the properties of identified proteins, the 1557 identified proteins were classified based on the cellular location or biological process of the protein using the GO database (**Figure 1**). Proteins that were not described or unspecified in the GO database were screened out and not further assessed for this report.

The melanosome is a tissue specific organelle containing melanin pigments. It is of interest to investigate how well the melanosomal proteins were extracted and identified in this study, since melanoma is associated with changes in pigmentation and melanosome biogenesis, and melanogenesis related proteins have been used as melanoma biomarkers [21], [22], [23]. Of the common melanosomal proteins 79 out of 100 were identified using our method. These common proteins are known as constituent and resident proteins of the melanosome. There are six well-known melanogenesis proteins that are also frequent signatures of melanomas: tyrosinase, TRP1/TYRP1, TRP2/DCT, gp100/pmel17, MART1, and GPR43/OA1. These proteins have been shown to play either an enzymatic or structural role in melanin synthesis [24], [25], [26]. Four out of six of these well-known melanosome-specific proteins found in cutaneous melanomas were identified in our proteomic profiling.

### Differentially Expressed Proteins in Metastasis

Of the 120 differentially expressed (q<0.05) proteins in priority 1, 61 had expression levels that differed by more than 20% between metastatic and primary melanoma (**Table 2**). A cut-off of 20% was selected based on a 15% median CV of the combined replicate and sample (**Table 1**). Of the 61 differentially expressed proteins, 38 were up-regulated and 23 were down-regulated.

**Table 2:** Metastasis compared to Primary Melanoma.

Accession number	Gene Symbol	Annotation	# of peptides sequenced	q-value
**Summary of Upregulated Proteins** *				
P62701	RPS4X	40S ribosomal protein S4, X isoform	2	0.003
P14786	PKM2	Pyruvate kinase isozymes M1/M2	20	0.003
Q8TBK5	RPL6	60S ribosomal protein L6	2	0.005
Q02878	GAPDHS	Glyceraldehyde-3-phosphate dehydrogenase, testis-specific	3	0.006
Q59GX2	GLUT1	Solute carrier family 2, facilitated glucose transporter member 1	2	0.007
Q14568	HSP90AA2	Heat shock protein HSP 90-alpha 2	17	0.008
P07195	LDHB	L-lactate dehydrogenase B chain	12	0.008
Q5RKT7	RPS27A	40S ribosomal protein S27a	4	0.011
P00354	GAPDH	Glyceraldehyde-3-phosphate dehydrogenase	24	0.012
Q6FHV0	MIF	Macrophage migration inhibitory factor	4	0.012
Q9BVK5	PPIB	Peptidyl-prolyl cis-trans isomerase B precursor	6	0.012
Q3B872	HIST1H2BI	Histone H2B type 1-C/E/F/G/I	10	0.013
Q3KQT6	RPS2	40S ribosomal protein S2	3	0.014
Q5J7W1	PGK1	Phosphoglycerate kinase 1	20	0.014
Q8WV32	TALDO1	Transaldolase	2	0.016
Q99497	DJ1	Protein DJ-1	8	0.016
Q9UBU5	PRDX5	Peroxiredoxin-5, mitochondrial precursor	4	0.016
Q76LA1	CSTB	Cystatin-B	3	0.016
Q53X54	HSPE1	10 kDa heat shock protein, mitochondrial	3	0.019
Q96DW8	SERPINA3	Alpha-1-antichymotrypsin precursor	2	0.02
Q9NTZ7	CPNE1	Copine-1	2	0.021
Q53Y44	PFN1	Profilin-1	6	0.021
Q8WU81	MYST4	Histone acetyltransferase MYST4	2	0.021
P05141	SLC25A5	ADP/ATP translocase 2	7	0.021
Q86WV2	COX4I1	Cytochrome c oxidase subunit 4 isoform 1, mitochondrial precursor	2	0.023
P12236	SLC25A6	ADP/ATP translocase 3	5	0.023
P42224	STAT1	Signal transducer and activator of transcription 1-alpha/beta	4	0.026
P31945	PRDX2	Peroxiredoxin-2	8	0.028
P07900	HSP90AA1	heat shock protein 90 kDa alpha	4	0.029
Q53ES3	ARL6IP5	PRA1 family protein 3	2	0.03
P14866	HNRPL	Heterogeneous nuclear ribonucleoprotein L	2	0.034
P13797	PLS3	Plastin-3	3	0.036
Q99623	PHB2	Prohibitin-2	6	0.04
Q9UDE9	LDHA	L-lactate dehydrogenase A chain	17	0.041
P16402	HIST1H1D	Histone H1.3	6	0.041
Q9H4B7	TUBB1	tubulin, beta 1	6	0.043
P02792	FTL	Ferritin light chain	5	0.047
P32077	PRDX6	Peroxiredoxin-6	4	0.047
**Summary of Downregulated Proteins** * **:**				
P33121	ACSL1	Long-chain-fatty-acid–CoA ligase 1	2	0.003
Q5T0R7	CAP1	Adenylyl cyclase-associated protein 1	4	0.003
P36957	DLST	Dihydrolipoyllysine-residue succinyltransferase component of 2-oxoglutarate dehydrogenase complex, mitochondrial precursor	2	0.003
Q53FQ6	CAPZA1	F-actin capping protein subunit alpha-1	2	0.004
P31930	UQCRC1	Ubiquinol-cytochrome-c reductase complex core protein 1, mitochondrial precursor	3	0.005
Q59EQ5	CSRP1	Cysteine and glycine-rich protein 1	2	0.006
P07585	DCN	Decorin precursor	2	0.007
P24941	CDK2	Cell division protein kinase 2	4	0.009
Q6LBZ1	APOE	Apolipoprotein E precursor	4	0.009
Q96QM7	LUM	Lumican precursor	6	0.009
Q9Y4L1	HYOU1	150 kDa oxygen-regulated protein precursor	4	0.011
Q504S5	CKAP4	Cytoskeleton-associated protein 4	3	0.012
P62241	RPS8	40S ribosomal protein S8	3	0.016
P78528	PRKDC	DNA-dependent protein kinase catalytic subunit	2	0.016
P05198	EIF2S1	Eukaryotic translation initiation factor 2 subunit 1	2	0.017
P15586	GNS	N-acetylglucosamine-6-sulfatase precursor	2	0.021
P41219	PRPH	Peripherin-2	3	0.021
Q9UKB0	HMGA1	High mobility group protein HMG-I/HMG-Y	2	0.022
Q3KQV4	NACA	Nascent polypeptide-associated complex subunit alpha	2	0.024
Q4V324	GAGE7	G antigen 7	3	0.026
P67809	YBX1	Nuclease sensitive element-binding protein 1	4	0.031
P13667	PDIA4	Protein disulfide-isomerase A4 precursor	4	0.034
Q16208	CD44	CD44 antigen precursor	2	0.042

* Proteins with significant changes greater than 20%.

**Figure 2:**
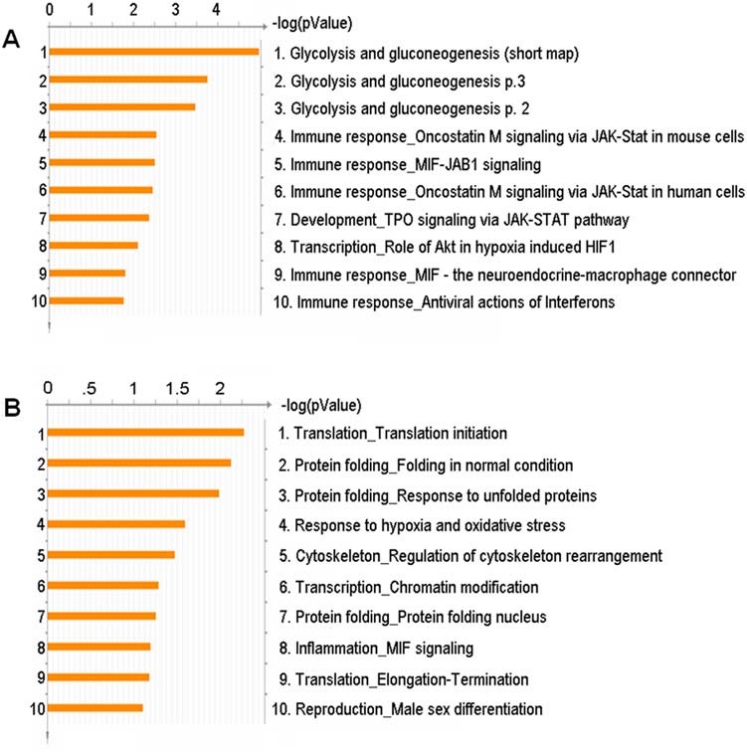
Representation of two ontological categories of differentially expressed proteins in metastasis by GeneGo pathways analysis. The results are ordered by −log10 of the p-value of the hypergeometric distribution. A) GeneGo canonical pathway maps, and B) GeneGo cellular process.

To further interpret the likely roles of differentially-expressed proteins involved in melanoma metastasis, we used the MetaCore™ pathway mapping tool to analyze and build the biological networks related to these proteins. First, we investigated the enrichment of the 61 differentially expressed proteins across two different ontological categories using GeneGo canonical pathway maps and GeneGo processes by calculating the hypergeometric distribution p-value of this list with respect to each category. **Figure 2A** shows the top ten GeneGo canonical pathway maps associated with the differentially expressed proteins and **Figure 2B** shows the top ten GeneGo cellular process networks. Glycolysis and gluconeogenesis, which involve PGK1, GAPDH, GAPDHS, PKM2, LDHA, and LDHB proteins, are suggested to have the highest significance in the pathway maps. Translation initiation and protein folding are the two most significant cellular process networks. Individual mapping of up-regulated and down-regulated proteins showed that glycolysis and gluconeogenesis were the most up-regulated pathways, while DNA damage-induced response (cell cycle), nucleocytoplasmic transport of CDK/cyclins (cell cycle), and DNA-damage-induced apoptosis were the most down-regulated (data not shown).

Next, we analyzed direct interaction between the 61 differentially expressed proteins. STAT1 directly interacted with HSP90AA1, SERPINA3, and APOE, whereas HMGA1 interacted with CD44. We then analyzed the transcription factors regulating 61 differentially expressed proteins. The most prominent regulatory proteins were c-MYC, SP1, and P53, interacting with 21, 20, and 11 differentially expressed proteins, respectively. Proteins directly activated by c-MYC included HSPE1, HSP90AA1, LDHA, CDK2, HMGA1, EIF2S1, and YBX1. Finally, we used the analyze network (AN) algorithm to reveal the most relevant networks associated with the 61 differentially expressed proteins. The networks were prioritized based on the number of fragments of the canonical pathways in the network. As a result, the top-scored AN network brought 11 proteins together: up-regulated proteins were MYST4, HIST1H2BI, PPIB, ARL6IP5, CPNE1, PFN1, and DJ1; downregulated proteins were YBX1, NACA, PRKDC, and CDK2.

**Figure 3:**
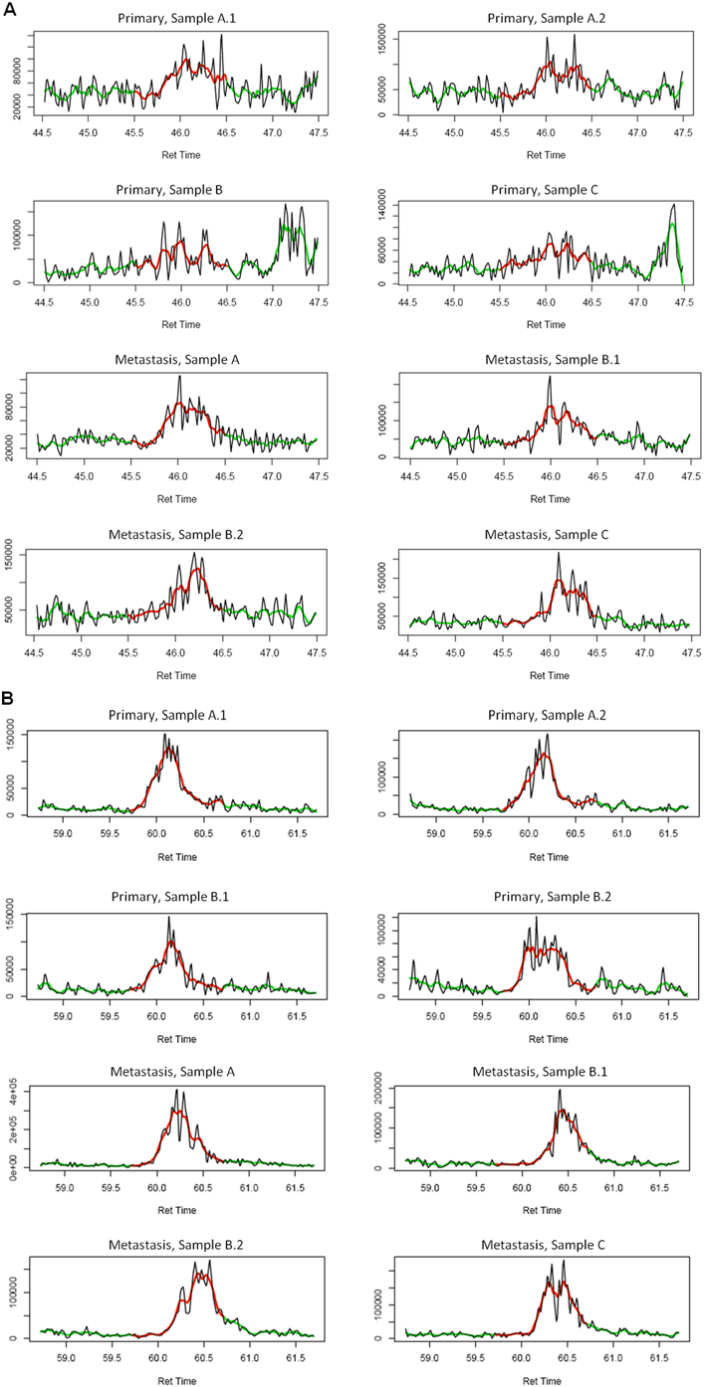
MS tracings of representative specific peptides. X-axis: retention time. Y-axis: peak intensity. A) Extracted ion chromatogram (XIC) for peptide GDYPLEAVR from pyruvate kinase isozymes M1/M2 of representative samples. Primary melanoma samples A.1 and A.2 are duplicate samples. Metastatic melanoma samples B.1 and B.2 are duplicates. Peak intensities were calculated by smoothing (as indicated by the green and red trace lines) and integrating the AUC of smoothed peaks for all samples within the same one-minute window (RT from 45.5 min to 46.5 min as indicated by the red trace line). B) Extracted ion chromatogram (XIC) for peptide ALLFVPR from heat shock protein HSP90AA2 of representative samples. Primary melanoma samples A.1 and A.2, and samples B.1 and B.2, are duplicate samples, respectively. Metastatic melanoma samples B.1 and B.2 are duplicates. RT from 59.7 min to 60.7 min as indicated by the red trace line.

**Figure 4:**
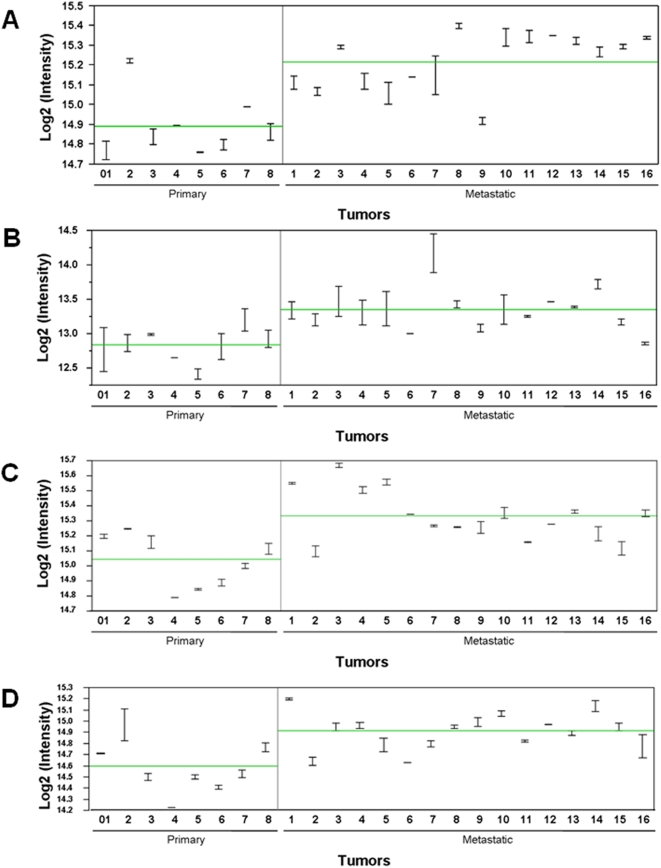
Representative plots of protein intensities in the individual samples of primary and metastatic melanoma. The individual protein intensities for A) PKM2, B) GLUT1, C) HSP90AA2, and D) LDHB Are plotted on a log_2_ scale. The horizontal line is the group mean, and duplicate intensities of individual samples are joined by a vertical line.

To identify the potential proteomic markers, our first objective was to focus on the up-regulated proteins that are categorized in the biological processes related to melanoma progression or metastasis (**Table 3**). These categories include cell structure and motility, cell adhesion, cell defense, cell cycle, cell proliferation and differentiation, apoptosis, metabolic process, and melanosome-related. Interestingly, all of the listed proteins have been previously implicated as cancer-related, and some have been suggested to be related to metastasis, including pyruvate kinase isozymes M1/M2 (PKM2), glucose transporter isoform 1 (GLUT1), macrophage migration inhibitory factor (MIF), peroxiredoxin-2 (PRDX2), L-lactate dehydrogenase A chain (LDHA), and heat shock protein HSP 90 alpha 2 (HSP90AA2). Furthermore, GLUT1, HSP90AA2, prohibitin-2 (PHB2), peptidyl-prolyl cis-trans isomerase B precursor (PPIB), and L-lactate dehydrogenase A chain (LDHA) have been identified in the melanosome. In **Figure 3** we provide representative MS tracings of XIC for specific peptides in PKM2 and HSP90AA2. **Figure 4** shows representative plots of protein intensities of the four most significant proteins are shown: PKM2 (q value = 0.003), GLUT1 (q = 0.007), HSP90AA2 (q = 0.008) and L-lactate dehydrogenase B chain (LDHB, q = 0.008) in individual samples of primary and metastatic melanomas.

**Table 3:** Selected Up-regulated Proteins in Melanoma Metastasis.

Category	Gene Symbol
**Cell Structure and Motility, Cell Adhesion**	TUBB1, PLS3
**Cell Cycle**	MYST4, PHB2, TUBB1
**Cell proliferation and differentiation**	DJ1, MYST4, PHB2
**Cell Defense**	MIF, PPIB, DJ1, PRDX5, PRDX2, PRDX6, HSP90AA2
**Carbohydrate metabolic process**	PKM2, GLUT1,LDHB, LDHA, PGK1
**Protein metabolic process**	HSP90AA1, HSP90AA2, HSPE1, MYST4, PPIB
**Nucleic acid metabolic process**	DJ1, MYST4, PHB2, STAT1
**Melanosome-Associated**	PKM2, GLUT1, HSP90AA1, PPIB, PHB2, LDHA, PLS3

### IHC Analysis of Proteins

**Figure 5:**
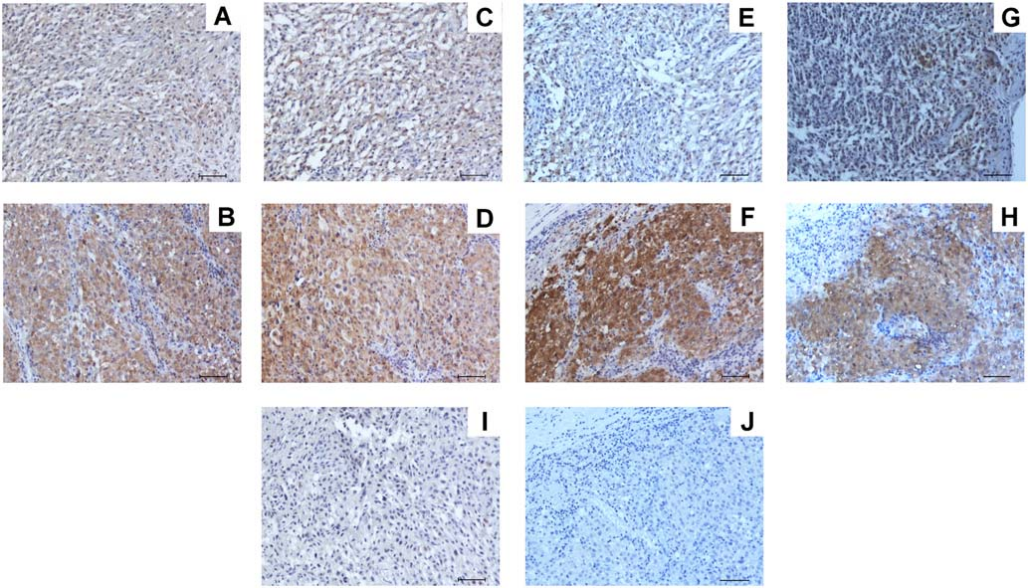
Representative IHC staining of significant proteins found in melanomas. Anti-PKM1/M2 (rabbit) Ab: A) primary and B) brain metastasis. Anti-HSP90 (rabbit) Ab: C) primary and D) brain metastasis. Anti-MIF (mouse) Ab: E) primary and F) brain metastasis. Anti-STAT1 (mouse) Ab: G) primary and H) brain metastasis. I) Universal negative control rabbit Ig on primary melanoma tissue. J) Universal negative control mouse Ig on brain metastasis tissue. All magnifications were ×100. Scale bars represent 100 µm.

We examined several key proteins that were significantly upregulated in metastasis compared to primary melanoma. IHC staining was limited to availability of antibodies for FFPE tissues. In **Figure 5** we show representative examples of IHC staining of PKM2, HSP90 alpha, MIF, and STAT1 in both primary and metastatic melanomas. These studies confirmed the MS analysis of proteins in melanomas.

## Discussion

Quantitative proteome analysis by MS has become a useful tool for understanding biological functions. While FFPE tissues offer great opportunity for retrospective proteomic analysis, isolating proteins from FFPE tissues suitable for MS analysis has been a challenge. Quality control and uniformity of preparation across, or even within, institutions remain problematic [27]. The reproducibility of results requires further validation on a large set of specimens.

In the present study, the global protein expression profile in FFPE tissue lysates of melanoma metastases was compared with that of primary tumors. Throughout this study, we have addressed several questions regarding FFPE melanoma tissues: (1) Is this methodology useful for profiling the protein expression of archival tissue? (2) How many proteins can be identified from small quantities of FFPE melanoma tissue using Liquid Tissue® reagents and high resolution LC-ESI/MS/MS? (3) Do specific identified proteins discriminate between metastatic and primary tissue? (4) Can known melanoma-related biomarkers be identified? (5) Are any known metastasis-related markers identified? (6) Can the identified protein be validated by other methods?

We have demonstrated that proteins can be extracted from microdissected FFPE tissues and utilized for MS analysis. Using LC/MS/MS analysis, over 1500 proteins can be identified in a small amount of cells (the equivalent of approximately 5,000 cells per injection). Moreover, identified proteins appear to cover a wide variety of biological functions and appear in diverse cellular compartments. The proteomic profiling of FFPE tissue demonstrated the detection of the major known melanogenesis pathway proteins that are known to be expressed in melanomas. Over 70% of previously known common melanosomal proteins can be identified, including four of the six well known melanoma specific markers. MART1 and TRP2 were not identified, which may either be due to their low abundance or interference from melanin [28], [29]. Melanin has been shown to bind to proteins, causing difficulties in protein solubility and binding affinity to chromatography columns, which impairs protein characterization using LC/MS analysis [30], [31]. It has been demonstrated that MART1 can be identified only if melanin is removed before 2D-LC/MS [29]. Lack of detection of some proteins may be due to the level of protein in the tissues. Of the 120 differentially expressed proteins identified with multiple peptides, 61 showed differences exceeding 20%. Many of these proteins have been reported to be associated with characteristic steps of metastasis including motility, adhesion, migration and tumor progression including cellular defense, apoptosis, and proliferation.

Several of the up-regulated proteins (**Table 3**) identified in this study have been described previously and implicated as cancer-related. Some proteins are implicated as metastasis-related proteins (e.g. LDH, HSP90, GLUT1, MIF, DJ1, PKM2 and PHB2). HSP90 and GLUT1 have been previously described in melanoma metastasis tissues as up-regulated [32], [33]. LDH protein levels have been described in serum from melanoma patients [34]. The expression of pyruvate kinase, HSP90 alpha, MIF, and STAT1 in primary and metastatic melanomas could be demonstrated by IHC staining. Upregulated expression levels of these proteins in melanoma metastasis compared to primary melanomas were demonstrated, confirming MS analysis.

In addition, LDHA, HSP90, GLUT1, MIF, DJ1, PKM2 PHB2, PGK1 and PPIB are secreted from cancer cells and can be detected in the blood, offering great potential as biomarkers of tumor progression. It may be interesting to investigate whether these proteins are involved in melanoma progression. Many of these proteins are poorly-documented, and their role in metastasis requires further investigation (e.g. MYST4, PLS3, and TUBB1). We were able to identify several proteins implicated in melanoma progression and metastasis; their biological functions and deregulations in tumorigenesis and metastasis are further discussed below.

Plastins are actin-binding proteins that have three cell-type specific isoforms: T-plastin, L-plastin and I-plastin. L-plastin is expressed in hemapoietic cells, while T-plastin is in cells of solid tissue, including fibroblasts, epithelial cells, and melanocytes. Upregulation of L-plastin has been well documented in many types of cancer [35] and has been implicated in metastasis [36]. However, the role of T-plastin in tumor progression is not known and overexpression of T-plastin in cultured fibroblasts showed increased mobility and altered cellular architecture. Although PLS3 (T-plastin) has not been reported to be specifically present in the melanosome organelle, it has been shown to be expressed in melanocytes [37].

HSP90 has been a potential target for cancer therapy in the last decade, and a HSP90 inhibitor, 17-allylamino-17 demethoxygeldanamycin (17-AAG), is currently in clinical trials [38]. HSP90 is involved in maintaining the conformation, stability, activity, and cellular localization of several key oncogenic client proteins, including ERBB2, C-RAF, CDK4, AKT/PKB mutated P53, hypoxia-inducible factor-1α (HIF-1α), survivin telomerase hTERT, and steroid hormone receptors [39]. Inhibition of HSP90 simultaneously targets multiple oncogenes and pathways, as well as characteristic traits of malignancy. HSP90α, an isoform of HSP90, is stress-inducible and overexpressed in many cancer cells [40]. Furthermore, HSP90α has been shown to be related to invasiveness and found to be secreted [40]. HSP90 mRNA levels are up-regulated in primary and metastatic melanoma when compared to melanocytic nevi [32]. Our analysis showed significantly higher levels of HSP90 protein in metastatic than in primary melanomas.

DJ1 protein was initially discovered as a putative oncogene that promotes cell survival by negatively regulating tumor suppressor PTEN [41]. DJ1 has also been implicated in cell protection against stresses including chemotherapy and oxidative stresses, [42]. Overexpression of DJ1 has been shown to be associated with tumorigenesis. Interestingly, DJ1 has been detected in serum from uveal malignant melanoma patients [43].

MYST4, a member of the MYST histone acetyltransferase family, may be involved in both upregulation and downregulation of tumorigenesis. MYST4 reportedly fuses to the transcription co-activators CBP, p300, and TIF2 in acute myeloid leukemia. MYST4 was shown to be required for RUNX2-dependent transcriptional activation, and to interact with P53 in cancer [44], [45]. There is little information about the expression profile of MYST4 in cancer. Further investigation is needed to determine whether MYST4 is associated with brain metastasis, since MYST4 has been implicated in neural development and maintenance of neural stem cells, and in this study we found MYST4 expression is higher in brain melanoma metastases when compared to primary melanoma.

Proteins of the prohibitin family (PHB1 and PHB2) are localized in many cellular compartments (e.g. mitochondria, nucleus, and cell-surface) and may have diverse functions in different locations. PHB appears to function as a chaperone in the stabilization of mitochondrial proteins [46], yet the functions of nuclear PHB are still controversial. PHB has been proposed to play a role as a potential tumor suppressor, but higher levels of PHB protein have been reported in a variety of cancers [47]. Prohibitin was demonstrated to play an unexpected role in the activation of the Ras-Raf signaling pathway and in modulating epithelial cell adhesion and migration, indicating that PHB may play a role in metastasis formation [47].

Several of the most significant proteins identified that increased in metastasis were those involved in the aerobic glycolysis pathway in cancer cells (Warburg effect). Particular upregulated proteins were GLUT1, PKM1/M2, PGK1, and LDHA/B. Most of these proteins have been shown to be upregulated in cancer, including melanomas. Interestingly, in our analysis these proteins of the glycolysis pathway were quite significantly elevated in metastatic tissues. LDHA and LDHB are subunits of lactate dehydrogenase (LDH) and associate to generate five tetrameric forms of LDH isoenzymes. LDHA is involved in metabolic activities, particularly the redox reaction at the end of glycolysis. An enhanced level of LDHA is observed in cancer cells and a higher level of LDHA is associated with proliferation [48]. Elevated serum levels of LDH indicate progression of AJCC stage IV melanoma; LDH is a biomarker for AJCC staging of melanomas [49]. Our results support the finding that upregulation of LDH in cells is related to aggressive metastatic melanoma tumors.

GLUT1 may be responsible for constitutive or basal glucose uptake. Overexpression of GLUT1 is associated with increased glucose metabolism and has been correlated with progression of several types of cancer [50]. Studies have shown that GLUT1 is modulated by hypoxia-responsive elements and upregulated by overexpression of HIF-1 [51]. GLUT1 protein is expressed in most melanoma metastases with a broad range of expression levels as shown by Western immunoblot analysis [33]. Our results confirm these findings.

PKM2 is an M2 type of pyruvate kinase (PK) which plays a critical role in aerobic glycolysis regulation in tumorigenesis. During oncogenesis, the PK isoform M1 is converted to PKM2 and is present as a less active dimer form. PKM2 is up-regulated in tumor cells, and is under control of Ras and transcription factors HIF-1, SP1, and SP2. Interestingly, tumor M2-PK is expressed at higher levels in metastases than in primary tumors, and the level of PKM2 in plasma of various cancer types is correlated with tumor size and stage [52]. Recent studies have shown that PKM2 isoform is highly necessary for aerobic glycolysis, which provides a selective growth advantage for tumor cells [53].

PGK1 (phosphoglycerate kinase), which is involved in the glycolytic pathway, is regulated by HIF-1α and is enhanced in states of hypoxia due to increased demand for glycolysis. PGK1 also affects DNA replication and repair in the nucleus. Extracellular secretion of PGK1 by tumor cells increases angiostatin activity, causing inhibition of angiogenesis. In several types of cancer, PGK1 has been shown to be up-regulated [54]. In pancreatic cancers, PGK1 is secreted into the bloodstream, and studies have shown increased levels of serum PGK1. Therefore, this protein shows promise as a potential serum marker for cancer [54].

In this report, Liquid Tissue® reagents effectively solubilized proteins and peptides from microdissected formalin-fixed melanoma tissues and enabled proteomics analysis by ESI-LC MS/MS. Global protein profiling using label-free protein quantitative analysis revealed potential metastasis-related proteins that are differentially expressed between metastatic and primary melanoma. Up-regulation of some of these proteins has been previously implicated in tumor progression by other methodologies and is in accordance with our findings. Hence, the method described here is extremely useful for proteomics studies in a high-throughput setting. Moreover, in the search for potential biomarker, candidates can be confirmed in a larger set of samples to validate the findings. The advantage of this approach is that it allows analysis of retrospective tumor tissue specimens with known disease outcomes. This provides a unique strategy to develop new molecular prognostic biomarkers of melanoma.
